# Wearable Smart Silicone Belt for Human Motion Monitoring and Power Generation

**DOI:** 10.3390/polym16152146

**Published:** 2024-07-28

**Authors:** Lijun Zhou, Xue Liu, Wei Zhong, Qinying Pan, Chao Sun, Zhanyong Gu, Jiwen Fang, Chong Li, Jia Wang, Xiaohong Dong, Jiang Shao

**Affiliations:** 1 The College of Mechanical Engineering, Jiangsu University of Science and Technology, Zhenjiang 212000, China; 212241802639@stu.just.edu.cn (L.Z.); zhongwei@just.edu.cn (W.Z.); 221110201207@stu.just.edu.cn (C.S.); jiawang@just.edu.cn (J.W.); 13952581050@126.com (X.D.); 2 The College of Chemistry and Molecular Sciences, Henan University, Kaifeng 475001, China; xliu@henu.edu.cn; 3 Department of Chemistry, Technical University of Denmark, DK-2800 Kongens Lyngby, Denmark; qipan@kemi.dtu.dk; 4 College of Chemical Engineering, Shijiazhuang University, Shijiazhuang 050035, China; gzy030201@163.com

**Keywords:** smart sensor, motion monitoring, energy collection, polymer

## Abstract

Human physical activity monitoring plays a crucial role in promoting personalized health management. In this work, inspired by an ancient Chinese belt, a belt-type wearable sensor (BWS) based on a triboelectric nanogenerator (TENG) is presented to monitor daily movements and collect the body motion mechanical energy. The developed BWS consists of a soft silicone sheet and systematically connected sensing units made from triboelectric polymer materials including polytetrafluoroethylene (PTFE) and polyamide (PA). A parameter study of the sensing units is firstly conducted to optimize the structure of BWS. The experimental studies indicate that the parameter-optimized BWS unit achieves a maximum output voltage of 47 V and a maximum current of 0.17 μA. A BWS with five sensing units is manufactured to record body movements, and it is able to distinguish different physical activities including stillness, walking, running, jumping, normal breathing, cessation of breathing, and deep breathing. In addition, the developed BWS successfully powers electronic devices including a smartphone, digital watch, and LED lights. We hope this work provides a new strategy for the development of wearable self-powered intelligent devices.

## 1. Introduction

The wearable device, which provides new opportunities to optimize human-computer interaction and improves health monitoring capabilities, has become a focal point of innovation in today’s ever-evolving technological landscape [1,2,3,4]. However, traditional wearable devices are characterized by their high cost and bulky designs, not to mention the frequent charging requirement [5,6]. Consequently, a self-powered wearable smart device that can be integrated with daily appliance is expected [7,8,9,10,11].

The TENG is foreseen as a key supplement to the next generation of wearable devices due to its advantages such as high instantaneous power outputs, efficiency, low cost, and ease of fabrication [12,13,14,15]. By combining contact electrification and electrostatic induction, TENGs can effectively convert mechanical energy into electrical energy [16,17,18]. The typical materials used in TENGs are polymeric materials due to their high stability, low cost, and diversity [19,20,21]. For instance, Yang et al. reported a contact-type polydimethylsiloxane (PDMS)-based TENG as a wearable power source, and the effects of human motion on output voltage, current, and power were investigated [22]. Chu et al. successfully developed a polymer-based TENG for self-powered wearable electronics [23]. The self-powered TENG fabricated with atomical thin graphene (<1 nm), PDMS (<1.5 µm), and polyethylene terephthalate (PET) (<0.9 µm) was applied in an assistive communication device and successfully transmitted Morse code. Huang et al. proposed a self-powered system for human physiological signal monitoring and energy collection [24]. The proposed system consisted of two low-cost and simply manufactured TENGs, which included Al and PDMS as the triboelectric materials. Li et al. proposed a bioinspired sweat-resistant wearable TENG for movement monitoring during exercise and fitness [25]. The reported TENG consists of two bioinspired superhydrophobic and self-cleaning triboelectric layers (elastic resin and PDMS), which featured the hierarchical micro/nanostructures replicated from lotus leaves. Although plenty of TENG-based wearable devices have been developed, it still remains challenge to integrate the wearable devices with daily appliance while maintaining their comfortability, sensibility, and energy collection capacity [26,27,28,29].

**Figure 1:**
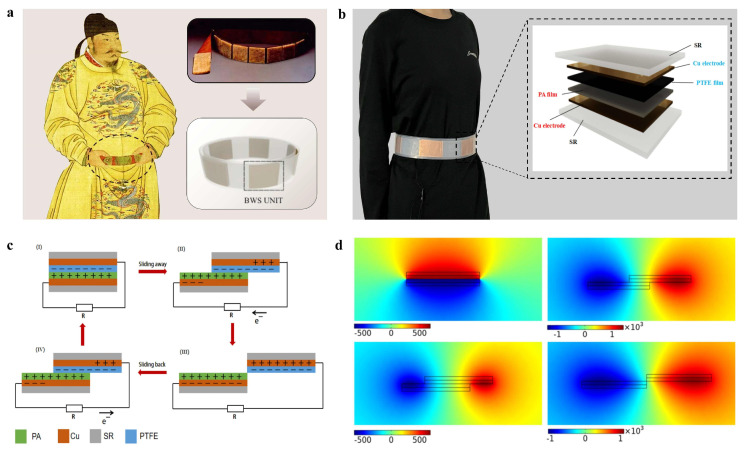
(**a**) The diagram of the BWS. (**b**) The photograph of the BWS worn on the waist. The inset is the enlarged view of the BTWS unit. (**c**) The freestanding working principle of the BWS follows steps (I–IV). (**d**) The potential distribution of the BWS at various displacements simulated with COMSOL Multiphysics 6.0.

In this work, we propose a belt-type wearable sensor that is inspired by an ancient Chinese belt to simultaneously realize the real-time monitoring of human movement and energy collection, as shown in Figure 1a. Different from previously reported wearable electronics, the developed BWS integrates the energy harvesting ability and sensing ability into the most common daily appliance-a belt-and is capable of simultaneously realizing power generation and recording human motion without imposing an extra burden upon users. PTFE and PA are employed in this study to serve as the triboelectric materials due to their accessibility, reliability, cost-effectiveness, and significant electronegativity difference. The effect of structural parameters of the BWS unit on the performance of the designed smart belt is evaluated under laboratory conditions. The experimental results indicate that the BWS unit can achieve a maximum output voltage of 47 V and a maximum current of 0.17 μA under experimental conditions with a sliding frequency of 0.5 Hz. Based on the optimized structure, BWSs with five sensing units are prepared and utilized to monitor different motion states. The results show that different physical activities including stillness, walking, running, jumping, normal breathing, cessation of breathing, and deep breathing can be successfully identified by the developed BWS. In addition, the energy harvesting ability of the proposed BWS is examined by powering an Apple smartphone, a digital watch, and 30 LEDs. We hope this study presents a new routine for developing daily articles’ integrated wearable devices with motion monitoring and power generation capacities.

## 2. Materials and Methods

### 2.1. Fabrication of BWS

Two triboelectric layers (width 40 mm, length 60 mm, and thickness 0.03 mm) including PTFE film and PA film are prepared, followed by covering their outer surfaces with Cu electrodes (width 40 mm, length 60 mm, and thickness 0.06 mm). PTFE films are purchased from Taizhou Chen Guang Plastic Industry Co., Ltd (Taizhou, China). PA films are purchased from Dongguan Fuxing Plastic Co., Ltd (Dongguan, China). The Cu electrodes are purchased from Huizhou Kesheng Industrial Co., Ltd (Huizhou, China). The BWS unit is manufactured by encapsulating the Cu-covered triboelectric layers in an SR sheet. The SR sheets are purchased from Shanghai Yuzhao Industrial Co., Ltd (Shanghai, China). The BWS (width 50 mm, length 850 mm, and thickness 10 mm) is prepared by arranging five uniformly distributed sensing units in two elastic PDMS membranes.

### 2.2. Electrical Measurement

The electrical signals are collected by a digital multimeter (Agilent 34401A, Agilent Technologies, Santa Clara, CA, USA).

## 3. Results and Discussion

As shown in Figure 1a, a wearable smart sensor based on a TENG, inspired by ancient Chinese belts, is proposed for smart monitoring as well as physical energy collection. Figure 1b shows the demonstration of the prepared wearable smart belt and an enlarged structure diagram of the BWS unit. The BWS unit employs polymer materials (PTFE and PA) as the triboelectric layers, while their outer surfaces are covered with copper (Cu) films as electrodes. The BWS units are systematically connected and arranged in the two silicone rubber (SR) sheets, forming the desired wearable smart belt. In the smart belt, one end of the inner silicone sheet is fixed while the other end remains free, which promises that the inner silicone sheet remains the same during respiration. With respect to the outer silicone sheet, both ends are fixed to make sure that it deforms with the expansion of the abdomen during respiration. Consequently, relative slipping is generated between two tribo-active materials during respiration. The working principle of the BWS is based on the triboelectrification and the electrostatic induction effect [30,31,32], as schematically illustrated in Figure 1c. In the original position (Figure 1c(I)), the surfaces of the PA film and PTFE film fully overlap and intimately contact with each other. As shown in Table S1, because of the significant difference in electron affinity between PA and PTFE, triboelectrification leaves the PA film surface with positive charges and the PTFE film with negative charges of equal density [33,34]. No charge transfers between the two electrodes due to the electrostatic equilibrium. Subsequently, with the relative slip between two triboelectric layers (Figure 1c(II)), charge transfer occurs due to the decrease in the contact surface area. Electrons transfer from the upper electrode to the lower electrode to maintain an electrostatic balance. The electron flow continues until the two surfaces are entirely separated (Figure 1c(III)). When the top film slides back (Figure 1c(IV)), electrons flow backward from the lower electrode to the upper electrode, generating a current opposite to the previous one until the two films completely overlap as the original position (Figure 1c(I)). As an effective method, the simulation is widely utilized to provide an in-depth understanding of the mechanism [35,36]. Hence, to demonstrate the working mechanism more clearly, the commercial software COMSOL Multiphysics 6.0 is used to simulate the corresponding electric potential distribution under four working states, as shown in Figure 1d. The modeling results show close agreement with the above analysis.

**Figure 2:**
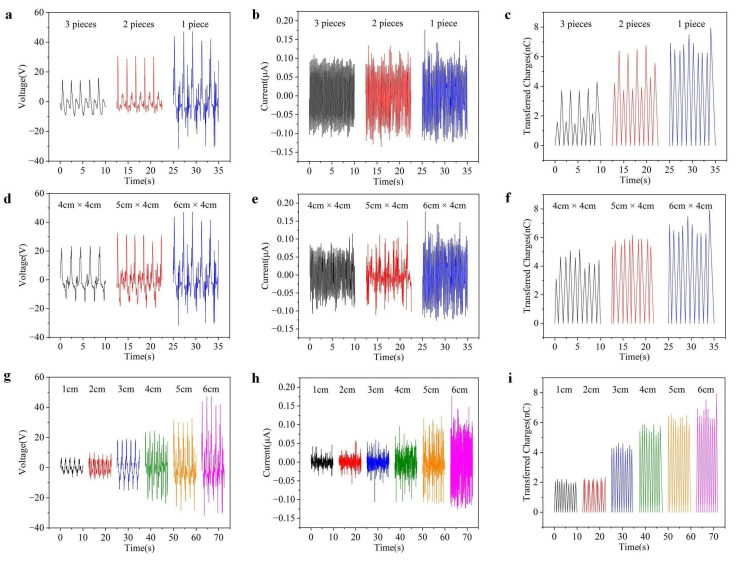
(**a**–**c**) The impact of the number of units on the electrical performance of the BWS, including (**a**) output voltage, (**b**) current, and (**c**) transferred charges. (**d**–**f**) The impact of the area on the BWS unit electrical performance, including (**d**) output voltage, (**e**) current, and (**f**) transferred charges. (**g**–**i**) The impact of the sliding distance on the electrical performance of the BWS unit, including (**g**) output voltage, (**h**) current, and (**i**) transferred charges.

To investigate the influence of structure parameters on the output performance of BWS, a sliding rail is utilized to fix the BWS unit and adjust the sliding distance and frequency. The impact of the number of divided pieces in a single BWS is explored with a sliding frequency of 0.5 Hz by keeping a constant contact area. The experimental results indicate that the maximum output voltage gradually increases from 16 V (three pieces) to 47 V (one piece) as the number of divided pieces decreases, and a similar tendency is also noticed in transferred charges as shown in Figure 2a,c. A single complete unit can effectively utilize the entire friction area to generate charge separation, which reduces the coupling effects and energy loss among multiple pieces. However, there is no significant difference in the output current as shown in Figure 2b, which may be attributed to the same total friction area in a single BWS unit. Apart from a divided piece number, the effect of the friction area on the output characteristics of BWS is examined by varying the unit sizes from 4 cm × 4 cm to 6 cm × 4 cm while keeping the piece number constant (one piece). The width of the BWS units is set to be 4 cm, the same as the commercial belt. As the area of the BWS unit increases, the output voltage, current, and transfer charge gradually increase, as shown in Figure 2d–f. Consequently, the length of the BWS unit in this design is set to be 6 cm × 4 cm. The impact of the sliding distance on the output of the BWS unit is investigated to simulate the effect of abdominal distension. As shown in Figure 2g–i, the output voltage, current, and transferred charge show an increasing trend as the sliding distance increases. This may be attributed to the increase in the effective power generation surface area [37,38,39].

**Figure 3:**
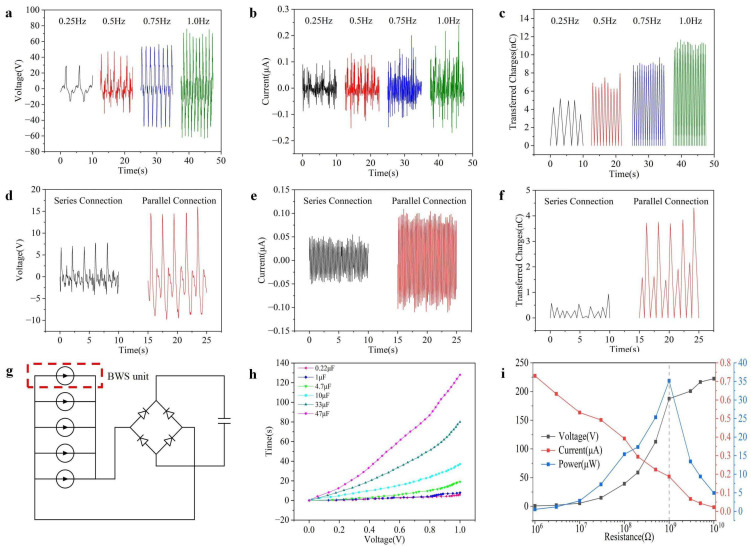
(**a**–**c**) The impact of sliding frequency on the electrical performance of the BWS unit, including (**a**) output voltage, (**b**) current, and (**c**) transferred charge. (**d**–**f**) Comparison of the electrical performance of the BWS between series and parallel connection, including (**d**) output voltage, (**e**) current, and (**f**) transferred charges. (**g**) The equivalent circuit of the BWS units connected in parallel. (**h**) Voltage curves of several commercial capacitors charged by the BWS. (**i**) The output voltage, current, and peak power of the BWS with 5 units under varied external load resistances.

The breathing rate varies among different people, and the respiratory rate is usually lower than 1 Hz. In this study, the effect of the breathing rate on the output of the BWS unit is investigated by varying the sliding frequency between 0.25 Hz and 1 Hz. Figure 3a–c illustrate the changes in the output voltage, current, and transferred charge of the BWS unit as a function of different sliding frequencies. It can be observed that the output voltage gradually increases with sliding frequency varying from 0.25 to 1 Hz. With respect to the current and transferred charge, they show an upward trend with the increase in sliding frequency. This can be explained by the increase in power generation cycles originating from frequency changes.

Previous studies have indicated a significant impact of the connection method between units on the output of TENGs [40,41,42]. In this study, the influence of the connection method on the output performance is evaluated by connecting three BWS units, as schematically illustrated in Figure 3d–f. The results indicate that the voltage, current, and transferred charge of parallel-connected BWS units are significantly higher than those of the series-connected BWS units, which is consistent with previous reports [43]. Therefore, the BWS with five parallel-connected BWS units is manufactured by integrating the units with SR with a length of 70 cm. An external rectifier bridge is utilized to convert alternating current to direct current, as shown in Figure 3g. To examine the power collection ability, the prepared BWS is integrated with an energy storage system to charge different capacitors, and the time required to charge each capacitor to 1 V is recorded to investigate the performance of the BWS. As shown in Figure 3h, the time required to charge the capacitor to 1 V gradually increases as the capacitance of the capacitor increases, and the capacitor with a capacitance of 4.7 μF can be charged to 1 V within 20 s. In addition, the electrical performance of the BWS is tested under different external load resistances from 10^6^ to 10^10^ Ω, as shown in Figure 3i. The experimental results show that a maximum power of 35 μW can be achieved under an external load resistance of 1 GΩ, which is obviously higher than previous studies, as illustrated in Figure S1.

**Figure 4:**
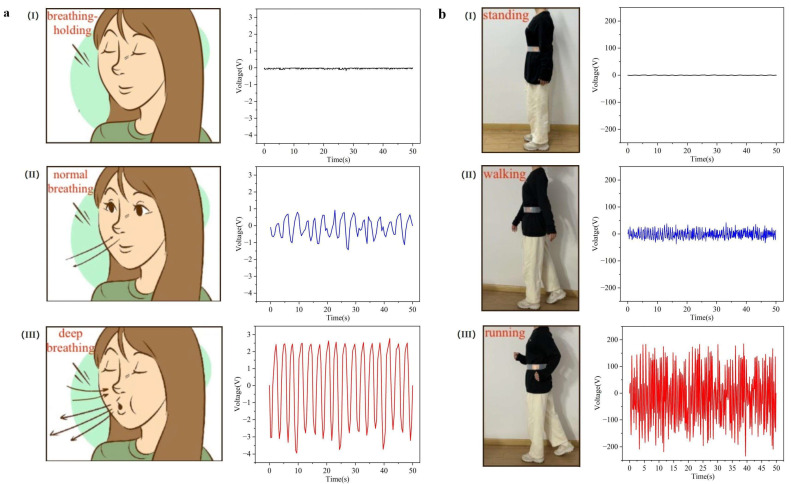
(**a-I**) The schematic diagram and output voltage of cessation of breathing. (**a-II**) The schematic diagram and output voltage of normal breathing. (**a-III**) The schematic diagram and output voltage of deep breathing. (**b-I**) The photograph of standing with the BWS and output voltage measured in a standing posture. (**b-II**) The photograph of walking with the BWS and output voltage measured in a walking posture. (**b-III**) The photograph of running with the BWS and output voltage measured in a running posture.

To demonstrate the sensibility of prepared BWS, it is used to monitor the different physical states during breathing and exercise. Figure 4a(I–III) are schematic diagrams of breath-holding, normal breathing, and deep breathing, respectively. When breathing is at rest, the output voltage of the BWS almost remains constant, as shown in Figure 4a(I). However, it is easy to notice that the electrical signals of normal breathing and deep breathing are significantly different from breath-holding, as illustrated in Figure 4a(II,III). Apart from different breathing states, the moving states including standing, walking, and running are monitored by the prepared BWS, and the electrical results are displayed in Figure 4b(I–III). The output of the BWS increases with the raise in exercise intensity and monitoring results can clearly distinguish standing, walking, and running states, which provides a judgment indicator for the integrity and effectiveness of the exercise. In addition, it is worth noting that various exercise parameters such as exercise time and frequency can also be obtained from the monitoring data, providing analysis and feedback for personal exercise monitoring and daily training. The long-term performance is one of the key indexes for wearable electronics. In this study, the long-term repeatability of the proposed BWS is examined by a stability test, as shown in Figure S2. The results show that no obvious deterioration is noticed after a 12 h test, demonstrating the reliability of the proposed BWS.

**Figure 5:**
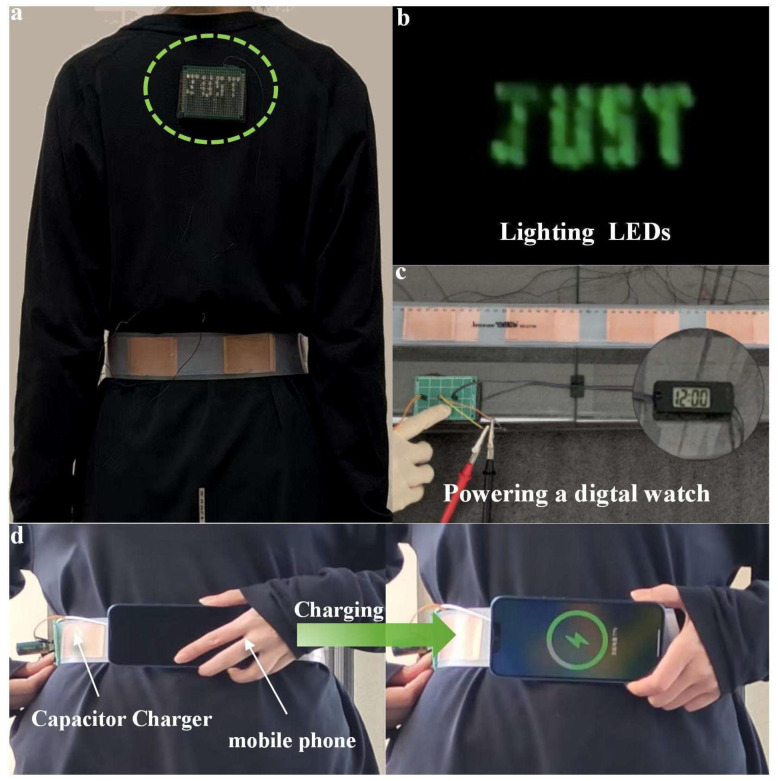
(**a**) The photograph of the green LEDs lighted by the BWS. (**b**) The green LEDs were lit up while starting running. (**c**) The photograph of the digital clock powered by the BWS. (**d**) The photograph of the wireless charging system based on the BWS.

The energy-harvesting abilities of wearable sensors can be used for establishing a self-powered system that sustainably drives wearable electronic products. To demonstrate this, the developed BWS worn by an experimenter is directly connected to the LED pattern “JUST” that consists of 30 LED lights without a capacitor, as shown in Figure 5a,b and Video S1. It can be observed that the LED pattern “JUST” is simultaneously illuminated once the experimenter starts to exercise. In addition, the energy generated by the proposed BWS is stored in a 10 μF capacitor and then utilized to power a digital watch, as shown in Figure 5c and Video S2. Eventually, we demonstrate the applicability of the developed BWS by collecting the energy generated from daily physical activities to charge a 1 F super capacitor, followed by successfully powering an Apple smartphone, as shown in Figure 5d and Video S3.

## 4. Conclusions

In summary, we propose a belt-type wearable smart sensor for daily movement monitoring as well as body motion energy collection. The proposed BWS is composed of five systematically connected sensing units and an SR sheet, which are manufactured from polymer materials such as PTFE, PA, and PDMS. The influence of the structural parameters on the output performance of the sensing units is firstly investigated and the experimental results indicate that a maximum output voltage of 47 V and a maximum current of 0.17 μA can be achieved by the parameter-optimized BWS unit. The sensibility of the designed BWS with five sensing units is successfully examined by recording different physical activities such as breathing, stillness, walking, and running. In the end, the energy collection abilities of the designed BWS are demonstrated by powering electronic devices including an LED pattern “JUST” (30 LED lights) and a digital watch as well as an Apple smartphone. We hope the proposed BWS provides an effective method for the development of wearable self-powered intelligent devices.

## Data Availability

The original contributions presented in the study are included in the article, further inquiries can be directed to the corresponding authors.

## Supplementary Materials

The following supporting information can be downloaded at https://www.mdpi.com/article/10.3390/polym16152146/s1, Figure S1: Comparison of our results with other published results; Figure S2: Stability test of the BWS; Table S1: Triboelectric series for some common materials; Video S1: Powering a digital watch; Video S2: The BWS drives LEDs by harvesting human movement; Video S3: Wireless charging system based on BWS. References [44,45,46,47,48] are cited in the supplementary materials.
