# Supplementary material for: Wearable Smart Silicone Belt for Human Motion Monitoring and Power Generation
Source: Polymers (Basel). 2024 Jul 28;16(15):2146. doi: 10.3390/polym16152146 (PMC11313891; doi:10.3390/polym16152146)
Supplement: Supplementary file 1 [file polymers-16-02146-s001.zip › polymers-3104240-supplementary.pdf]

Supporting information

Wearable Smart Silicone Belt for Human Motion Monitoring and Power Generation

Lijun Zhou[1], Xue Liu[2], Wei Zhong[1], Qinying Pan[3], Chao Sun[1], Zhanyong Gu[4], Jiwen Fang[1], Chong Li[1], Jia Wang[1], Xiaohong Dong[1] and Jiang Shao[1*]

This file includes:

Table S1. Triboelectric series for some common materials.

Figure S1. Comparison of our results with other published results.

Figure S2. Stability test of the BWS.

1 The College of Mechanical Engineering, Jiangsu University of Science and Technology, Zhenjiang 212000, China; jiangshao@just.edu.cn

2 The College of Chemistry and Molecular Sciences, Henan University, Kaifeng 475001, China; xliu@henu.edu.cn

3 Department of Chemistry, Technical University of Denmark, DK-2800 Kongens, Lyngby, Denmark; qi-pan@kemi.dtu.dk

4 College of Chemical Engineering, Shijiazhuang University, Shijiazhuang 050035, China; gzy030201@163.com

* Correspondence: jiangshao@just.edu.cn

	Aniline-formol resin	Polyvinyl alcohol	
	Polyformaldehyde 1.3-1.4	Polyester (Dacron) (PET)	
	Etylcellulose	Polyisobutylene	
Positive	Polyamide 11	Polyuretane flexible sponge	
	Polyamide 6-6	Polyethylene terephthalate	
	Melanime formol	Polyvinyl butyral	
	Wool, knitted	Formo-phenolique, hardened	
	Silk, woven	Polychlorobutadiene	
	Polyethylene glycol succinate	Butadiene-acrylonitrile copolymer	
	Cellulose	Nature rubber	
	Cellulose acetate	Polyacrilonitrile	
	Polyethylene glycol adipate	Acrylonitrile-vinyl chloride	
	Polydiallyl phthalate	Polybisphenol carbonate	
	Cellulose (regenerated) sponge	Polychloroether	
	Cotton, woven	Polyvinylidine chloride (Saran)	
	Polyurethane elastomer	Poly(2,6-dimethyl polyphenyleneoxide)	
	Styrene-acrylonitrile copolymer	Polystyrene	
	Styrene-butadiene copolymer	Polyethylene	
	Wood	Polypropylene	
	Hard rubber	Polydiphenyl propane carbonate	
	Acetate, Rayon	Polyimide (Kapton)	
	Polymethyl methacrylate (Lucite)	Polyethylene terephtalate	Negative
	Polyvinyl alcohol	Polyvinyl Chloride (PVC)	
	(continued)	Polytrifluorochloroethylene	
		Polytetrafluoroethylene (Teflon)	

Table S1. Triboelectric series for some common materials following a tendency to easily lose electrons (positive) and to gain electrons (negative) [1].

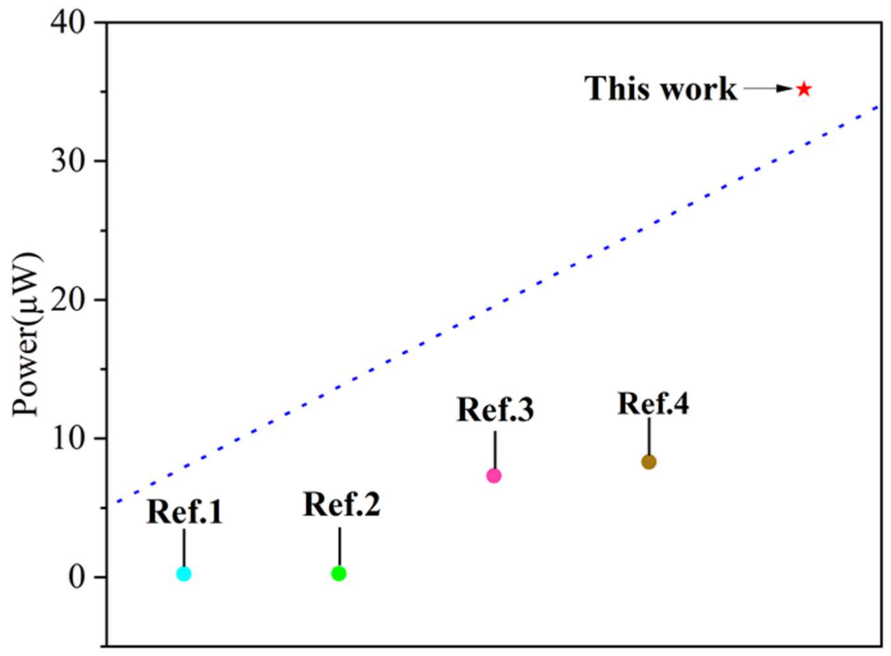

Figure S1. Comparison of our results (output power) with other published results, Ref. 1 (the self-powered TENG sensor) [2], Ref. 2 (the SLG-TENG) [3], Ref. 3 (the WP-TENG) [4], and Ref. 4 (the wearable PyNG) [5].

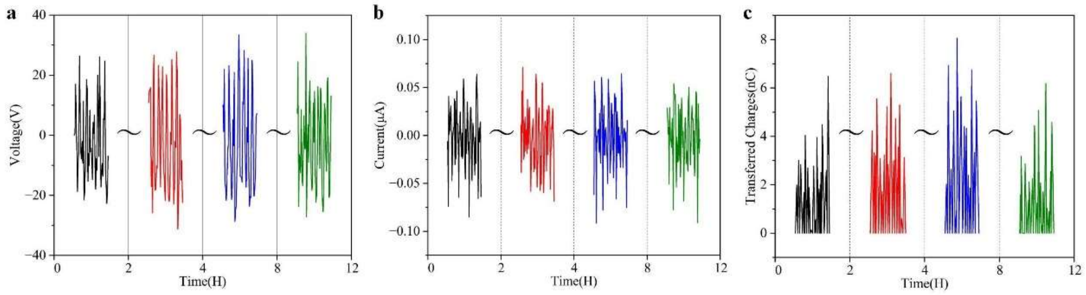

Figure S2. Stability test of the BWS.

Reference

1. Wang Z L. Triboelectric nanogenerators as new energy technology for self-powered systems and as active mechanical and chemical sensors. *ACS Nano* **2013**,7 (11), 9533–9557.
2. Zhang H, Zhang J, Hu Z, et al. Waist-wearable wireless respiration sensor based on triboelectric effect. Nano Energy 2019, 59: 75-83.
3. Jo S, Kim I, Jayababu N, et al. Antibacterial and soluble paper-based skin-attachable human motion sensor using triboelectricity. ACS Sustainable Chemistry & Engineering 2020, 8(29), 10786-10794.
4. Wen Z, Yang Y, Sun N, et al. A wrinkled PEDOT: PSS film based stretchable and transparent triboelectric nanogenerator for wearable energy harvesters and active motion sensors. Advanced Functional Materials 2018, 28(37), 1803684.
5. Xue H, Yang Q, Wang D, et al. A wearable pyroelectric nanogenerator and self-powered breathing sensor. Nano Energy 2017, 38, 147-154.
